# Infantile peripheral neuropathy, deafness, and proximal tubulopathy associated with a novel mutation of the RRM2B gene

**DOI:** 10.3325/cmj.2013.54.579

**Published:** 2013-12

**Authors:** Vesna Stojanović, Johannes A. Mayr, Wolfgang Sperl, Nenad Barišić, Aleksandra Doronjski, Gordana Milak

**Affiliations:** 1 Faculty of Medicine, University of Novi Sad, Novi Sad, Serbia; 2 Institute for Child and Youth Health Care of Vojvodina, Novi Sad, Serbia; 3 Department of Pediatrics, Paracelsus Medical University, Salzburg, Austria

## Abstract

Mitochondrial DNA depletion syndromes are a group of autosomal recessive hereditary disorders characterized by reduction of the amount of mitochondrial DNA in the affected tissue (muscle, liver, brain, or kidneys). We report a case of an infant with myopathy, deafness, peripheral neuropathy, nephrocalcinosis, proximal renal tubulopathy, moderate lactic acidosis, and a novel mutation of the RRM2B gene.

Mitochondrial DNA depletion syndromes are a group of autosomal recessive hereditary disorders characterized by reduction of the mitochondrial DNA amount in the affected tissue (1). Depletion of mitochondrial DNA can affect specific tissues or combination of organs and tissues including muscles, liver, brain, or kidneys (2,3).

Different defects of nuclear genes may lead to different clinical manifestations, such as hepatocerebral syndrome, encephalopathy, or myopathy. One of the recently identified genes for mitochondrial DNA depletion syndromes is RRM2B, which encodes an isoform of a small subunit of ribonucleotide reductase. This enzyme plays an essential role in nucleotide synthesis, converting ribonucleotides to deoxyribonucleotides. Since 2008, 14 mutations of *RRM2B* gene have been reported (3,4). All the reported mutations are unique and there is no mutation that appears in more than one family (1-4).

All reported patients had myopathy and primary lactic acidosis. More than a half of them died before the fourth month of age. The oldest patient with RRM2B mutation was a 42 years old woman with clinical findings suggestive of neurogastrointestinal encephalopathy (5). In this report, we review a case of an infant with muscular hypotonia, myopathy, peripheral neuropathy, deafness, nephrocalcinosis, proximal renal tubulopathy, moderate lactic acidosis, and a novel mutation of the *RRM2B* gene.

## Case report

The patients was a male Caucasian baby, born by spontaneous vaginal delivery at 38 weeks of gestation, (birth weight 2950 g, birth length 50 cm, Apgar scores 10/10). He was the second child of non-consanguineous parents. The elder child was healthy. The mother had well-controlled hypothyreosis due to thyroidectomy performed 12 years earlier and since then she had been using hormone replacement therapy (levothyroxine sodium). Family history was negative for genetic and hereditary diseases. During the first two months of life while he was at home, the baby had poor appetite and failed to thrive.

On admission, at the age of 3 months, he had growth delay: body weight 3800 g (3rd percentile), body length 58 cm (25th percentile), and head circumference 38 cm (3rd percentile). Except for the generalized hypotonia (“floppy infant”) and inability to completely close the left eyelid, all other physical findings were normal.

**Figure 1:**
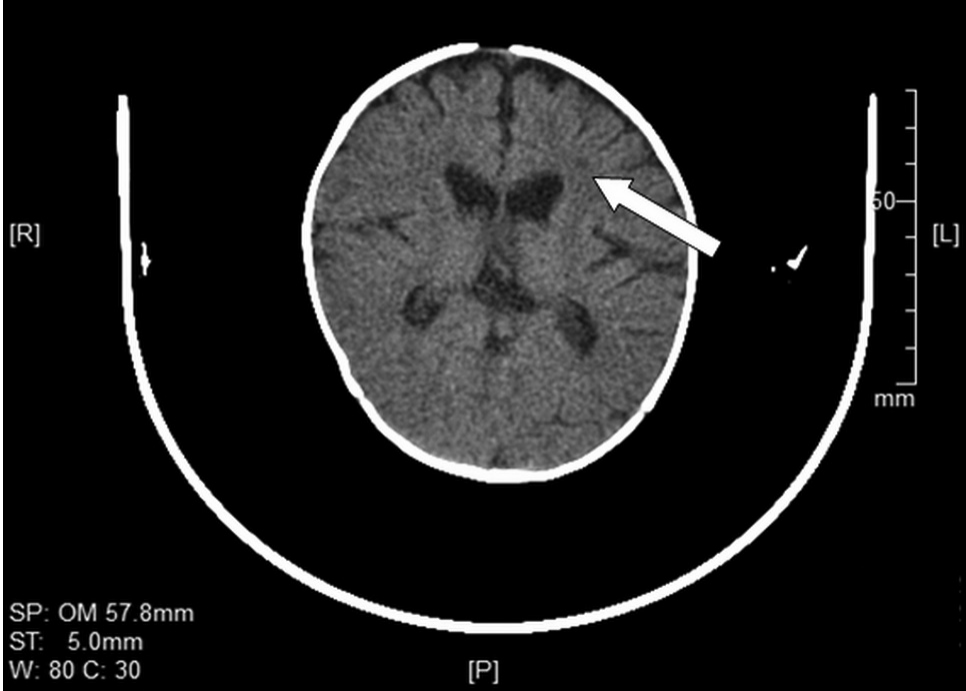
Computed tomography scan of the brain showing microlacunar hypodense zones in periventricular white matter of the frontal lobes.

Complete blood count and other biochemical findings (routine kidney and liver function tests), including creatine phosphokinase, were within the reference range. Plasma lactate levels were slightly elevated and ranged from 2.0 mmol/L to 8.0 mmol/L (reference range 0.1-2.2 mmol/L). Urinary calcium/creatinine ratio ranged from 2.9 mmol/L to 8.1 mmol/mmol (upper reference value 2.2 mmol/mmol). Parathyroid hormone was 13.2 pg/mL (15-65 pg/mL) and vitamin D 142 mmol/L (30-100 mmol/L). Tubular reabsorption of renal phosphate per deciliter glomerular filtrate (TP/GFR) was 0.41. Plasma levels of amino acids – threonine, valine, cystine, beta alanine, histidine, arginine, and praline were significantly reduced. The results of urine analysis showed: pH 7.0, specific gravity 1000, proteins 3+, glycosuria 55.5 mmol/L, microalbuminuria 26.3 to 300 mg/L (upper reference value 16 mg/L), albumin/creatinine ratio >20.3 mg/mmol (upper reference value 1.4 mg/mmol). Concentrations of alanine, isoleucine, leucine, ornithine, and lysine in urine were significantly elevated. Electrocardiography and echocardiography findings were normal. Ultrasonography of the urinary tract showed that both kidneys were of normal shape and size, with hyperechogenic renal pyramids. Computed tomography of the abdomen showed calcium deposits in the renal pyramids of both kidneys. Brain ultrasound was normal. Computed tomography of the brain (Figure 1) revealed microlacunar hypodense zones in periventricular white matter of the frontal lobes that corresponded to ischemic lesions. Both lateral ventricles, as well as the third ventricle, were slightly dilated. Subarrachnoid spaces in the Sylvian fissures were moderately expanded on both sides (Figure 1).

Electroencephalography showed non-specific electrocortical dysrhythmia. Electromyography and nerve conduction velocity tests revealed neurogenic lesion of mild to moderate degree on all tested muscles of upper and lower extremities, more expressed distally. Denervation activity was observed in all tested muscles. Neurographic parameters pointed to a deceleration of transmission for all tested motor nerves and lowered amplitudes for axillar and median nerves on the left.

Ophthalmologic investigations revealed a megacornea (right eye 12.5 mm, left eye 12 mm) on both sides. Anterior eye segment and fundus were normal. Transient-evoked otoacoustic emission did not register any response. There was a doubt that it could be the case of sensorineural deafness with a hearing limit lower than 35 dB Hearing Level.

Muscle biopsy (musculus vastus lateralis) revealed no signs of atrophy, inflammation, and fibrosis. Vacuolar myopathy was seen on all intersections. The specimens were dyed using periodic acid-Schiff staining method and the findings were negative. Aggregation of mitochondria was seen only in some fibers. Pathognomic “ragged red” fibers were not seen. Neural cell adhesion molecule was expressed on sarcolemma and in cytoplasm, but only a few fibers indicated expression on the neuromuscular connection.

Soon after admission to the intensive care unit, pneumonia with complete atelectasis of the left lung was diagnosed. Mechanical ventilation was initiated due to respiratory failure, along with appropriate antibiotic therapy. Further clinical course was progressive and complicated with recurrent lung infections and sepsis. Despite of the hypercaloric enteral intake, he progressively lost body weight. His psychomotor skills continuously deteriorated and he developed progressive external ophthalmoplegia. The patient died at the age of 8 months.

**Figure 2:**
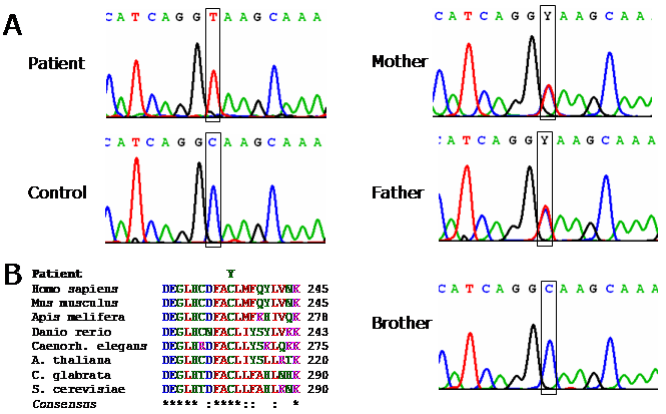
Sequence analysis revealed a mutation c.707G>A,p.Cys236Tyr in RRM2B affecting a phylogentically highly conserved amino acid.

The mother and the patient had normal karyotypes – 46, XX and 46, XY, respectively; both with pericentric inversion of the chromosome 9 (p11q13). The father had male karyotype (46, XY) with deletion of one copy of exon 7 SMN1 gene. Investigation of the *RRM2B* gene from genomic DNA isolated from blood of the patient revealed a missense mutation c.707G>A, p.Cys236Tyr, which affected a highly conserved amino acid. The mutation allele was the only detectable, making a homozygous mutation likely (Figure 2).

## Discussion

There are three forms of mitochondrial DNA depletion syndromes – myopathic, encephalomyopatic, and hepatocerebral (3). The incidence of mitochondrial encephalopathy in children is about 1 per 5000-11 000 newborns. Mitochondrial disease is caused by genetic defects of mitochondrial DNA or nuclear genes encoding proteins of the respiratory chain enzyme complexes. Replication of mitochondrial DNA is regulated by nuclear genes, encoding nucleotide supply, as well as mitochondrial DNA replication process. The lactate level in plasma is moving from normal to significant lactic acidosis (6).

Depletion of mitochondrial DNA is associated with mutations in the nuclear genes that affect metabolism of mitochondrial nucleotides and replication of mitochondrial DNA. These genes exert their action by either directly affecting the mitochondrial DNA replication fork (POLG, POLG2, PEO1) or regulating the mitochondrial deoxynucleotide triphosphate pool (TYMP, TK2, DGUOK, SUCLA2, SUCLG1, ANT1). In patients with mitochondrial depletion syndrome, a novel mutation of RRM2B was diagnosed, which presents with early muscular hypotonia, renal tubulopathy, diarrhea, lactic acidosis, and rapid progressive lethal course (3).

All the patients with autosomal recessive RRM2B mutations described until now have had myopathy and most of them have had renal involvement. The disease usually begins during infancy with myopathy and lactic acidosis (3,4). In our patient, plasma lactate levels shifted from initially normal values up to 8 mmol/L.

So far, congenital deafness has been described only in 3 patients with RRM2B gene mutation (3,6). In our patient, transient-evoked otoacoustic emission showed no response. Brain imaging findings have been reported in only 2 patients. One had bilateral diffused reduction of white matter and the other generalized atrophy of the brain (6,7). Our patient also had mild abnormalities of the brain (ventriculomegalia). Electromyography and nerve conduction velocity tests revealed neurogenic lesions on tested muscles on all extremities. Denervation activity was registered in all tested muscles. Motor nerve conduction study showed deceleration of transmission velocity for all tested nerves. This is an important finding because until now peripheral neuropathy has not been described in patients with RRM2B mutation, but only in patients with POLG mutation (7). Mitochondrial encephalomyopathy is important in differential diagnosis of neonatal muscular hypotonia (Table 1).

**Table 1:** Clinical and genetic features of reported cases with RRM2M mutations

No	Reference number		Clinical features	CT/MRI of the brain	CK	Serum lactate	EMG	Skeletal muscle biopsies	Molecular genetic analysis
1	1	Family 1	Total of 3 subjects; a brother and two sisters, all with same clinical features: trunk hypotonia, proximal tubulopathy	NA	NA	++	NA	Severely decreased malate + glutamate oxidation and complex IV deficiency	Homozygous mutation (nt 850 C>T)
2									
3									
4		Family 2	Subject 1: trunk hypotonia, proximal tubulopathy, seizures	NA	NA	++	NA	Combined complex I, III, and IV deficiency	Compound heterozygote for a splice-site mutation (IVS3-2 A>G) and missense mutation (nt 580 G>A)
5			Subject 2: trunk hypotonia, proximal tubulopathy						
6		Family 3	Subject 1: respiratory distress, hypotonia	NA	NA	++	NA	Few RRFs and lack of histochemical cytochrome c oxidase reaction in all fibers	Compound heterozygote for two missense mutations (nt 190 *t* > C, W64R, and nt 581 A>G,E194G)
7			Subject 2: Trunk hypotonia, vomiting, diarrhea	NA	NA	++	NA	RRFs, COX deficiency	3-bp inframe deletion (nt 253-255ΔGAG, ΔGlu85) and a missense mutation (nt 707 G>T, C236F)
8	2		Deafness, progressive weakness, poor head control, persistent diarrhea, respiratory distress	Spectroscopy showed the presence of lactate in the left basal ganglia^†^	normal	++	NA	RRFs with modified Gomori trichrome stain and ragged-blue fibers with the SDH stain. Lipid accumulation	Homozigous for a c.671 *t* > G mutation in exon 6
9			Failure to thrive, failure to gain developmental milestones, hypotonia, microcephaly, respiratory failure, urinary infections, intolerance to oral feeds, peripheral pigmentary retinopathy	Bilateral and nearly symmetrical non-enhancing areas of abnormal signal reduced diffusion in the white matter. Spectroscopy showed lactate peak in the basal ganglia and CSF^‡^	normal	+	NA	Scattered fibers with increased staining for SDH	Compound heterozygous for a missense mutation in exon 8 (c.846 G>C) and 1-bp deletion in exon 9 (c.920 delA)
10			Progressive hypotonia, failure to thrive and microcephaly, multiple respiratory infections	NA	+	+	NA	Scattered RRFs COX negative or COX deficient	Compound heterozygous for a missense mutation in exon 9 (c.949 *t* > G) and 1-bp deletion in exon 6 (c.584 delG)
11	3		Floppiness, congenital deafness, glycosuria	NA	+	++	Neurogenic lesion	RRFs COX negative, lipid accumulation	c.G122C p.R41P / c.G122C p.R41P
12			Floppiness, tubulopathy	NA	NA	++	NA	NA	IVS3-2A>C / c.C328T p.R110C
13	4	brothers	Feeding difficulties, failure to thrive, severe muscular hypotonia, seizures, proximal tubulopathy	NA	normal	++	NA	COX deficiency and accumulation of fat in muscle fibers	Missense mutation in exon 7 (c.686 G>T)
14			Feeding difficulties, failure to thrive, severe muscular hypotonia, seizures	Generalized atrophy^‡^	++	++	NA	COX deficiency and accumulation of fat in muscle fibers	Missense mutation in exon 7 (c.686 G>T)
15	Our case		Myopathy, deafness, peripheral neuropathy, nephrocalcinosis, proximal renal tubulopathy	Microlacunar hypodense zones in periventricular white matter of the frontal lobes, slightly dilated lateral ventricles and third ventricle, subarachnoid spaces in the Sylvian fissures moderately expanded^†^	normal	+	Neurogenic lesion of mild to moderate degree	Vacuolar myopathy, aggregation of mitochondria in some fibers. No RRFs	Missense mutation c.707 G>A, p.Cys236Tyr

*Abbreviations: CK – creatine phosphokinase; EMG – electromyography; RRF – ragged red fibers; COX – cyclooxygenase, SDH – succinate dehydrogenase; + – moderately elevated levels, ++ – significantly elevated levels, NA – data not reported.

†Computed tomography (CT) of the brain.

‡Magnetic resonance imaging (MRI) of the brain.

It is still unknown why mutations are manifested only on specific organs when they are expressed ubiquitously. The reason for this could be the short lifetime of these patients, which prevents us from detecting changes in other organs. So far, mutation has been manifested in all patients as an early developed myopathy and in most of them as renal involvement. Our patient had myopathy, congenital deafness, nephrocalcinosis, proximal tubulopathy, and lactic acidosis. Peripheral neuropathy has still not been described in any of the other patients with RRM2B mutation. In our patient, the disease had a progressive course with fast psychomotor delay, progressive external ophthalmoplegia, failure to thrive, and respiratory insufficiency.
